# Association between long-term exposure to air pollution and the risk of incident laryngeal cancer: a longitudinal UK Biobank-based study

**DOI:** 10.1007/s11356-023-26519-y

**Published:** 2023-03-28

**Authors:** Jiada Wang, Chen Lin, Yidian Chu, Hongxia Deng, Zhisen Shen

**Affiliations:** Department of Otorhinolaryngology Head and Neck Surgery, Lihuili Hospital Affiliated to Ningbo University, Ningbo, Zhejiang China

**Keywords:** Laryngeal cancer, Air pollution, Genetic risk score, Prospective study

## Abstract

We assessed the association between long-term joint exposure to ambient air pollutants and the risk of laryngeal cancer and whether this risk was modified by genetic susceptibility. We used a multivariable Cox proportional hazards regression model to analyze data from UK Biobank to determine the relationship between long-term exposure to air pollutants–nitric oxide (NO), nitrogen dioxide (NO_2_), and 2.5-*µ*m and 10-*µ*m particulate matter (PM_2.5_ and PM_10_) and the risk of laryngeal cancer. In multivariable-adjusted models, in model 3 and compared with the participants with lower quintile scores for air pollution, the participants with the highest quintile scores for air pollution had a higher laryngeal cancer risk. The observed association was more pronounced among the participants who were female, were smokers, had a systolic blood pressure equal to or greater than 120 mmHg, and had diabetes. Compared with the participants with a low GRS and the lowest quintile score for air pollution exposure, those with an intermediate GRS and the highest quintile score for air pollution exposure had a higher risk of laryngeal cancer. Long-term exposure to NO_2_, NO, or PM_2.5_, individually or jointly, was associated with a risk of incident laryngeal cancer, especially in the participants with an intermediate GRS.

**Supplementary Information:**

The online version contains supplementary material available at 10.1007/s11356-023-26519-y.

## Introduction

Laryngeal cancer is one of the most common and invasive malignant tumors of the head and neck, and has an annual incidence worldwide of approximately 2.4% (Christensen et al. 2013). The pathogenesis of laryngeal cancer has not been fully determined but is regarded as related to many factors, such as long-term excessive smoking, heavy drinking, viral infection, exposure to gaseous chemicals or severe air pollution, and/or long-term exposure to radionuclides (Edefonti et al. 2010).

This study focuses on the effect of air pollution on laryngeal cancer, given that air pollution—especially that due to the main atmospheric pollutants, such as nitrogen dioxide (NO_2_), nitric oxide (NO), and 2.5-µm and 10-µm particulate matters (PM_2.5_ and PM_10_)—is recognized as having adverse effects on human health (Khaniabadi et al. 2017). Exposure to air pollution is significantly related to an increased risk of lung cancer, bladder cancer, kidney cancer, and urinary tract cancer (Zare Sakhvidi et al. 2020); however, little is known about the effect of air pollution on the risk of laryngeal cancer in the general population. Genetic factors are also closely related to the pathogenesis of laryngeal cancer (Stanaway et al. 2018) and may interact with many environmental factors contributing to its pathogenesis (Schraufnagel et al. 2019). However, it remains unknown whether a genetic predisposition alters the association between combined exposure to various air pollutants and the risk of laryngeal cancer.

We used data from UK Biobank (UKB) to comprehensively investigate the association between PM_2.5_, PM_10_, NO_2_, and NO exposure, an air pollution score based on these pollutants, and the risk of laryngeal cancer among middle- and older-aged adults. Moreover, we explored whether the relationship between air pollution exposure and laryngeal cancer risk was affected by genetic susceptibility.

## Methods

### Study population and data sources

UKB is a large prospective cohort with more than 500,000 participants recruited during 2006–2010. At recruitment, the detailed information of each participant (such as their body measurements, health status, medical diagnosis, family history, and lifestyle behaviors) was collected using standard sociodemographic questionnaires. The UKB study was approved by the Northwest Research Ethics Committee (ref: 11/NW/0382), and all of its participants have provided written informed consent. The current study used UKB resources (application number 78619).

The initial sample of the current study was 502,490. We excluded participants with laryngeal cancer at baseline or who were missing data on exposure to any air pollutants. The final analytic sample comprised 418,914 participants. The detailed flow diagram used for participant inclusion is shown in supplementary Fig. 1.

### Definition of the genetic risk score

Detailed information about genotyping, data imputation, and quality control in the UKB study has been described previously (Bycroft et al. 2018). We used a weighted method to calculate a genetic risk score (GRS) for laryngeal cancer using eight single-nucleotide polymorphisms (SNPs) that have been identified in genome-wide association studies (Supplementary Table 1) (Kampa and Castanas 2008). That is, the score of each SNP was determined by the number of risk alleles carried by an individual (0, l, or 2), and then the individual’s GRS for laryngeal cancer was calculated by the following weighted method: GRS = (*β*1 × SNP1 + *β*2 × SNP2 + ... + *β*8 × SNP8) × (8/sum of the *β* coefficients). We classified participants into tertiles based on their GRS: a low tertile (tertile 1), an intermediate tertile (tertile 2), and a high tertile (tertile 3).

### Definition of outcome

Laryngeal cancer was defined according to code C32 in the International Statistical Classification of Diseases and Related Health Problems (10th Revision). Participants were followed from enrolment until the time of laryngeal cancer diagnosis or censoring, with the latter defined as the time of death, withdrawal from the study, or the end of follow-up, whichever came first.

### Definition of the air pollution score

The annual average concentrations of NO_2_, NO, PM_10_, and PM_2.5_ were estimated using a land-use regression model developed from the European Study of Cohorts for Air Pollution Effects project and linked to participants’ residential addresses given at baseline (Cornean et al. 2019). The land-use regression model was calculated according to the data from European Study of Cohorts for Air Pollution Effects performed between January 26, 2010 and January 18, 2011. The spatial visualization mapping of geographic location information has been previously published [Obesity and the relation between joint exposure to ambient air pollutants and incident type 2 diabetes: a cohort study in UK Biobank] (Li et al. 2021).

We created an air pollution score to capture joint exposure to various air pollutants, which was calculated as follows: (*β* PM_2.5_ × PM_2.5_ + *β* PM_10_ × PM_10_ + *β* NO_2_ × NO_2_ + *β* NO × NO) × (4/sum of the *β* coefficients). The higher the air pollution score, the higher the degree of exposure to ambient air pollution. The *β* coefficient was obtained from the final model with each air pollutant considered (i.e., one at a time) as the independent variable.

### Definition of covariates

The baseline questionnaire collected data on the participants’ age, sex (male/female), ethnicity (White/non-White), work (employed/unemployed), education level (0–7 years, 8–10 years, 11–15 years, or 16 or more years), income level (< 18,000; 18,000–52,000; 52,000–100,000; > 100,000 pounds, £), smoking status, and alcohol consumption status (current, previous, or never). In addition, body-mass index (BMI) was calculated by dividing the weight (in kg) by the square of the height (m^2^), using the height and weight data at baseline. Systolic blood pressure (SBP) was the average of two automatic or manual measurements at baseline, as measured by professionally trained nurses. Hypertension and diabetes were diagnosed based on participants’ self-reported and medical record data. Metabolic equivalent task (MET) was defined as minutes per week for all activities including walking, moderate, and vigorous activity.

### Statistical analysis

Continuous variables are expressed as means ± standard deviations, and categorical variables are presented as percentages. Participants were stratified into quintiles based on air pollution exposure, with the first quintile as the reference.

We devised an air pollution score to assess joint exposure to NO_2_, NO, PM_10_, and PM_2.5_, which is determined by summing each pollutant concentration weighted by its regression coefficients with laryngeal cancer obtained from single-pollutant models. A multivariable Cox proportional risk model was used to estimate the risk ratio (hazard ratio (HR)) and 95% confidence interval (CI) for the relationship between each pollutant or air pollution score and laryngeal cancer. Model 1 was adjusted for age, sex, and ethnicity (White/non-White); model 2 was additionally adjusted for work (employed/unemployed), education (0–7 years, 8–10 years, 11–15 years, or 16 or more years), income (< 18,000; 18,000–52,000; 52,000–100,000; > 100,000), physical activity (continuous), alcohol-drinking status (never/past/current), and smoking status (never/past/current); model 3 was additionally adjusted for BMI (continuous), SBP (continuous), hypertension at baseline (yes/no), and diabetes at baseline (yes/no). Trends were estimated by regarding quintiles as continuous variables (i.e., 1–5), and restricted cubic splines were used to examine the relationship between each pollutant, air pollution scores, and the risk of laryngeal cancer (Licitra et al. 2003).

We also calculated the weighted GRS of laryngeal cancer to evaluate whether the genetic susceptibility of laryngeal cancer modified the association between the air pollution score and laryngeal cancer incidence. Thus, we tested the gene–air pollution interaction by adding variable cross-product terms of air pollution score with GRSs for laryngeal cancer into the models. Then, the presence of effect modification was examined with respect to sex (men vs. women), age (< 60 vs. ≥ 60 years), BMI (< 25 kg/m^2^ vs. ≥ 25 kg/m^2^), smoking status (no vs. yes), alcohol consumption status (no vs. yes), diabetes (no vs. yes), and SBP (< 120 mmHg vs. ≥ 120 mmHg). Sensitivity analyses were also conducted by excluding participants who had (Christensen et al. 2013) developed laryngeal cancer within the past 2 years, or (Edefonti et al. 2010) lived at their current address for fewer than 5 years.

All analyses were performed using SAS software (version 9.4; SAS Institute Inc., Cary, NC, USA) and R software (version 3.5.1). All ***p***-values for the tests were two sided, and ***P***-values < 0.05 were considered as statistically significant.

## Result

The baseline characteristics of the participants sorted according to the quintile of the air pollution score are shown in Table 1. The means ± standard deviations of air pollution score in the first to fifth quintiles were 44.7 ± 3.1, 52.1 ± 1.5, 56.6 ± 1.2, 61.1 ± 1.5, and 71.6 ± 8.2, respectively. Compared with participants in the lowest quintile for air pollution score, those in the highest quintile for air pollution score were younger, more likely to be male, non-White, current smokers, employed, have a lower education (0–7), have a low income (< 18,000), have hypertension, and have diabetes. The air pollutants were highly correlated with each other, and their Pearson correlation coefficients are shown in Supplementary Table 2.

**Table 1:** Baseline characteristics of the UK Biobank participants according to the quintiles of air pollution score (*N* = 418,914)

	Quintiles of air pollution score				
	Q1	Q2	Q3	Q4	Q5
Follow-up duration (person-years)	12.6 (1.6)	12.6 (1.7)	12.5 (1.7)	12.5 (1.7)	12.5 (1.8)
Age, years	57.5 (7.8)	57.3 (8.0)	57.0 (8.1)	56.4 (8.2)	55.7 (8.3)
BMI, kg/m^2^	27.1 (4.5)	27.4 (4.7)	27.6 (4.8)	27.7 (4.9)	27.6 (5.1)
Met, minutes per week	2720.7 (2732.3)	2641.3 (2689.9)	2670.1 (2735.5)	2680 (2763.2)	2668.8 (2743.6)
Systolic blood pressure, mmHg	140.1 (21.2)	139.6 (21.1)	139.4 (21.2)	139.1 (21.7)	138.2 (22.5)
Diastolic blood pressure, mmHg	83.6 (13.0)	83.4 (12.9)	83.4 (13.2)	83.7 (13.6)	83.7 (14.5)
NO_2_, μg/m^3^	17.2 (2.6)	22.6 (2.1)	26.3 (2.2)	30.0 (2.3)	37.4 (6.1)
NO, μg/m^3^	26.8 (4.4)	36.3 (3.7)	42.4 (3.7)	48.7 (4.2)	66.3 (17.2)
PM_10_, μg/m^3^	14.5 (1.8)	15.9 (1.5)	16.4 (1.4)	16.8 (1.5)	17.6 (1.8)
PM_2.5_, μg/m^3^	8.7 (0.4)	9.5 (0.3)	10.0 (0.3)	10.4 (0.4)	11.5 (0.9)
Air pollution score	44.7 (3.1)	52.1 (1.5)	56.6 (1.2)	61.1 (1.5)	71.6 (8.2)
Sex					
Female	44,739 (53.4)	44,698 (53.3)	44,765 (53.4)	44,505 (53.1)	44,188 (52.7)
Male	39,044 (46.6)	39,085 (46.7)	39,018 (46.6)	39,278 (46.9)	39,594 (47.3)
Ethnicity					
White	82,252 (98.2)	80,897 (96.6)	79,400 ()	76,572 (91.4)	72,658 (86.7)
Others	1531 (1.8)	2886 (3.4)	4383 (5.2)	7211 (8.6)	11,124 (13.3)
Work					
Unemployed	35,816 (42.7)	35,598 (42.5)	34,902 (41.7)	33,269 (39.7)	31,986 (38.2)
Employed	47,967 (57.3)	48,185 (57.5)	48,881 (58.3)	50,514 (60.3)	51,796 (61.8)
Education, years					
0–7 years	10,757 (12.8)	13,491 (16.1)	15,091 (18.0)	15,876 (18.9)	15,816 (18.9)
8–10 years	13,753 (16.4)	15,314 (18.3)	15,580 (18.6)	14,930 (17.8)	12,830 (15.3)
11–15 years	16,435 (19.6)	15,627 (18.7)	14,747 (17.6)	13,928 (16.6)	12,810 (15.3)
16 years	42,838 (51.1)	39,351 (47.0)	38,365 (45.8)	39,049 (46.6)	42,326 (50.5)
Income (USD)					
< 18,000	14,102 (16.8)	18,164 (21.7)	20,755 (24.8)	22,997 (27.4)	26,655 (31.8)
18,000–52,000	43,530 (52.0)	44,591 (53.2)	43,854 (52.3)	42,325 (50.5)	38,462 (45.9)
52,000–100,000	20,057 (23.9)	17,133 (20.4)	15,758 (18.8)	14,988 (17.9)	13,837 (16.5)
> 100,000	6094 (7.3)	3895 (4.6)	3416 (4.1)	3473 (4.1)	4828 (5.8)
Drinking status					
Never	2434 (2.9)	3108 (3.7)	3839 (4.6)	4602 (55.4)	5610 (6.7)
Previous	2191 (2.6)	2621 (3.1)	2948 (3.5)	3201 (3.8)	3858 (4.6)
Current	79,158 (94.5)	78,054 (93.2)	76,996 (91.9)	75,980 (90.7)	74,314 (88.7)
Smoking status					
Never	48,712 (58.1)	47,792 (57.0)	46,444 (55.4)	45,708 (54.6)	42,429 (50.6)
Previous	28,837 (34.4)	28,805 (34.4)	28,950 (34.6)	28,423 (33.9)	28,883 (34.5)
Current	6234 (7.4)	7186 (8.6)	8389 (10.0)	9652 (11.5)	12,470 (14.9)
Hypertension					
No	62,488 (74.6)	61,121 (73.0)	60,660 (72.4)	60,430 (72.1)	60,713 (72.5)
Yes	21,295 (25.4)	22,662 (27.0)	23,123 (27.6)	23,353 (27.9)	23,069 (27.5)
Diabetes					
No	80,502 (96.1)	79,750 (95.2)	79,454 (94.8)	78,954 (94.2)	78,744 (94.0)
Yes	3281 (3.9)	4033 (4.8)	4329 (5.2)	4829 (5.8)	5038 (6.0)

Data for continuous variables are presented as mean ± SD. Data for categorical variables are presented as *n* (%)

*BMI*, body mass index; *MET*, metabolic equivalent task; *NO*, nitric oxide; *NO*_*2*_, nitrogen dioxide; *PM*, particulate matter

**Fig. 1:**
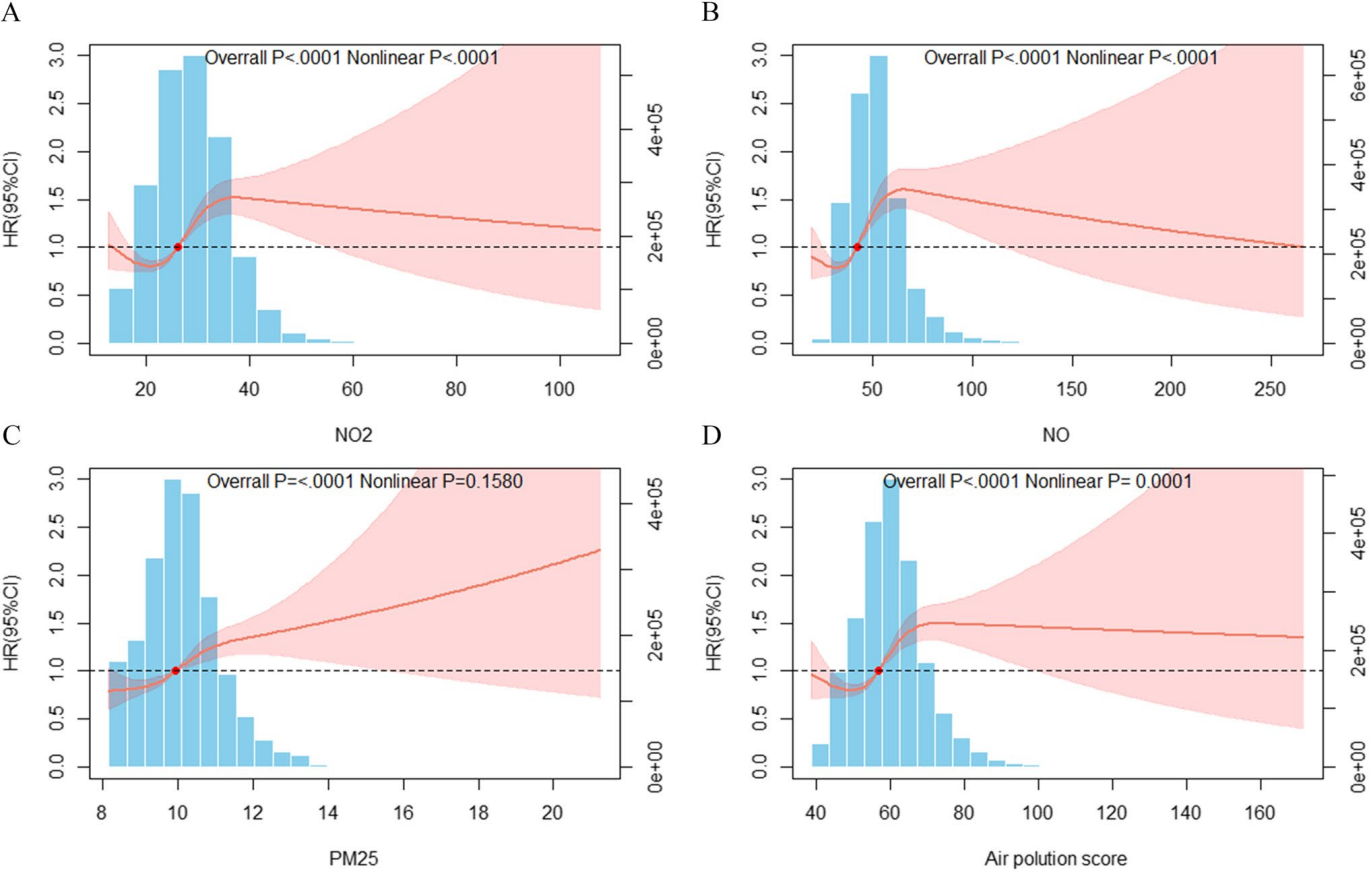
Associations of air pollutants (individually or jointly) with risk of laryngeal cancer. Cox proportional hazards models were used for analysis, including restricted cubic spline term for each air pollutant, with adjustment for age, sex, ethnicity (White/non-White), work (employed/unemployed), education (0–7 years, 8–10 years, 11–15 years, or 16– years), income (< 18,000/18,000–52,000/52,000–100,000/ > 100,000), physical activity (continuous), drinking status (never/past/current), smoking status (never/past/current), BMI (continuous), SBP (continuous), hypertension at baseline (yes/no), and diabetes at baseline (yes/no)

In the follow-up (a median follow-up period of 12.5 years), 1200 cases of laryngeal cancer occurred in 418,914 participants. The relationship between each air pollutant and the risk of laryngeal cancer is shown in Table 2. In model 3, we found that compared with the participants in the lowest quintile for exposure to NO_2_, NO, and PM_2.5_, the HRs of participants exposed to the highest quintile of the air pollutants NO_2_, NO, and PM_2.5_ were 1.7 (95% CI: 1.13–2.54), 2.13 (95% CI: 1.39–3.27), and 1.85 (95% CI: 1.20–2.85), respectively. A spline analysis showed a J-shaped relationship between exposure to NO_2_ and NO and the risk of laryngeal cancer (all *p*-nonlinearity < 0.001), but a linear association between exposure to PM_2.5_ and the risk of laryngeal cancer (*p*-linearity < 0.001) (Fig. 1). The associations between air pollution scores and the risk of developing laryngeal cancer are shown in Table 3. Compared with air pollution scores in the lower quintiles, air pollution scores in the highest quintile were found to be significantly associated with a higher risk of laryngeal cancer. In model 3 and compared with participants with an air pollution score in the lowest quintile, participants with an air pollution score in the highest quintile had a higher risk of laryngeal cancer [HR: 1.99 (95% CI: 1.30–3.03, *P* for trend = 0.001]. The spline analysis showed an S-shaped relationship between air pollution score and the risk of laryngeal cancer (*p*-nonlinearity < 0.001) (Fig. 1).

**Table 2:** HRs of laryngeal cancer by individual air pollutants among 418,914 UK Biobank participants

	Quintiles of air pollutants					HR (95% CI) per SD increase	*P* for trend
	Q1	Q2	Q3	Q4	Q5		
NO_2_, μg/m^3^							
Event	38	32	50	46	74	-	-
Person year	1,057,695	1,052,382	1,051,933.298	1,046,019.513	1,041,902.753	-	-
Model 1	Ref	0.86 (0.54, 1.37)	1.39 (0.91, 2.12)	1.33 (0.87, 2.05)	2.37 (1.6, 3.52)	1.30 (1.24, 1.36)	
*P*		0.522	0.128	0.190	0.001	0.001	0.001
Model 2	Ref	0.78 (0.49, 1.25)	1.18 (0.77, 1.8)	1.07 (0.69, 1.65)	1.7 (1.14, 2.55)	1.18 (1.12, 1.24)	
*P*		0.301	0.448	0.759	0.009	0.001	0.001
Model 3	Ref	0.78 (0.49, 1.25)	1.18 (0.77, 1.81)	1.07 (0.60, 1.65)	1.7 (1.13, 2.54)	1.18 (1.12, 1.24)	
*P*		0.303	0.444	0.757	0.010	0.001	0.001
NO, μg/m^3^							
Event	31	41	42	48	78	-	-
Person year	1,056,391	1,052,359	1,046,467	1,045,746	1,048,970	-	-
Model 1	Ref	1.36 (0.85, 2.17)	1.44 (0.91, 2.3)	1.71 (1.09, 2.69)	2.98 (1.96, 4.52)	1.24 (1.20, 1.29)	
*P*		0.194	0.120	0.019	0.001	0.001	0.001
Model 2	Ref	1.26 (0.79, 2.00)	1.24 (0.77, 1.97)	1.38 (0.88, 2.18)	2.14 (1.40, 3.27)	1.16 (1.11, 1.21)	
*P*		0.339	0.375	0.164	0.001	0.001	0.000272
Model 3	Ref	1.26 (0.79, 2.01)	1.24 (0.78, 1.97)	1.39 (0.88, 2.19)	2.13 (1.39, 3.27)	1.16 (1.11, 1.21)	
*P*		0.337	0.369	0.161	0.001	0.001	0.001
PM_10_, μg/m^3^							
Event	36	51	43	57	53	-	-
Person year	1,062,568	1,054,989	1,058,837	1,029,075	10,44,463	-	-
Model 1	Ref	1.44 (0.94, 2.21)	1.25 (0.80, 1.95)	1.76 (1.16, 2.68)	1.6 (1.04, 2.44)	1.09 (1.04, 1.15)	
*P*		0.092	0.325	0.007	0.030	0.001	0.018
Model 2	Ref	1.34 (0.87, 2.05)	1.1 (0.71, 1.72)	1.52 (1.00, 2.31)	1.36 (0.89, 2.08)	1.04 (0.98, 1.1)	
*P*		0.180	0.664	0.050	0.159	0.215	0.134
Model 3	Ref	1.34 (0.87, 2.05)	1.1 (0.71, 1.72)	1.51 (1.00, 2.30)	1.36 (0.89, 2.08)	1.04 (0.98, 1.1)	
*P*		0.180	0.666	0.052	0.159	0.208	0.136
PM_2.5_, μg/m^3^							
Event	31	40	51	48	70	-	-
Person year	1,054,234	1,064,892	1,047,761	1,039,018	1,044,027	-	-
Model 1	Ref	1.32 (0.83, 2.11)	1.75 (1.12, 2.74)	1.7 (1.08, 2.68)	2.6 (1.7, 3.97)	1.29 (1.23, 1.36)	
*P*		0.246	0.013	0.021	0.001	0.001	0.001
Model 2	Ref	1.21 (0.76, 1.93)	1.55 (0.99, 2.42)	1.38 (0.88, 2.18)	1.85 (1.2, 2.85)	1.16 (1.1, 1.22)	
*P*		0.430	0.056	0.162	0.005	0.001	0.004
Model 3	Ref	1.21 (0.76, 1.94)	1.55 (0.99, 2.42)	1.39 (0.88, 2.19)	1.85 (1.2, 2.85)	1.16 (1.1, 1.22)	
*P*		0.426	0.055	0.160	0.005	0.001	0.004

Model 1 was adjusted for age, sex, ethnicity (White/non-White); model 2 further adjusted for work (employed/unemployed), education (0–7 years, 8–10 years, 11–15 years, or 16– years), income (< 18,000/18,000–52,000/52,000–100,000/ > 100,000), physical activity (continuous), drinking status (never/past/current), smoking status (never/past/current); model 3 was additionally adjusted for BMI (continuous), SBP (continuous), hypertension at baseline (yes/no), and diabetes at baseline (yes/no)

**Table 3:** Associations between air pollution score and incident laryngeal cancer among 461,191 UK Biobank participants

	Quintiles of air pollution score					HR (95% CI) per SD increase	*P* for trend
	Q1	Q2	Q3	Q4	Q5		
Event	32	37	50	45	76	-	-
Person year	10,55,764	1,051,702	1,046,987.401	1,045,506.648	1,049,972.324	-	-
Model 1	Ref	1.19 (0.74, 1.91)	1.67 (1.07, 2.60)	1.57 (1.00, 2.47)	2.8 (1.85, 4.24)	1.29 (1.23, 1.35)	
*P*		0.477	0.023	0.051	0.001	0.001	0.001
Model 2	Ref	1.09 (0.68, 1.75)	1.43 (0.92, 2.24)	1.26 (0.80, 2)	1.99 (1.3, 3.04)	1.17 (1.12, 1.24)	
*P*		0.719	0.114	0.314	0.001	0.001	0.001
Model 3	Ref	1.09 (0.68, 1.75)	1.43 (0.92, 2.24)	1.27 (0.80, 2.00)	1.99 (1.3, 3.03)	1.17 (1.11, 1.23)	
*P*		0.717	0.113	0.311	0.001	0.001	0.001

Model 1 was adjusted for age, sex, ethnicity (White/non-White); model 2 further adjusted for work (employed/unemployed), education (0–7 years, 8–10 years, 11–15 years, or 16– years), income (< 18,000/18,000–52,000/52,000–100,000/ > 100,000), physical activity (continuous), drinking status (never/past/current), smoking status (never/past/current); model 3 was additionally adjusted for BMI (continuous), SBP (continuous), hypertension at baseline (yes/no), and diabetes at baseline (yes/no)

Stratified analyses revealed that sex, BMI, SBP, smoking status, drinking status, and diabetes significantly modified the association between air pollution score and the risk of laryngeal cancer (all *p* interactions < 0.01), with an overall HR of 0.85 (95% CI: 0.74–0.97), and individual HRs as follows: for participants who were female (HR = 3.73, 95% CI: 1.86–7.49), had an SBP equal to or greater than 120 mmHg (HR = 1.97, 95% CI: 1.61–2.41), were smokers (HR = 2.37, 95% CI: 1.64–3.42), or had diabetes (HR = 5.36, 95% CI: 2.10–13.69).

We also assessed the effect of the joint association between air pollution score and GRS for laryngeal cancer on the risk of laryngeal cancer (Table 4). Compared with participants with a low GRS and an air pollution score in the lowest quintile, those with an intermediate GRS and an air pollution score in the highest quintile had a higher risk of laryngeal cancer (HR = 2.64 (95% CI: 1.82–3.81); *p*-interaction = 0.001). Moreover, those with an intermediate GRS and NO_2_, NO, and PM_2.5_ scores in the highest quintile had the highest risk of laryngeal cancer.

**Table 4:** The joint association of the air pollutants (individually or jointly) and laryngeal cancer genetic risk score with the risk of incident laryngeal cancer in model 3

	Quintiles of air pollutants					*P* for interaction
	Q1	Q2	Q3	Q4	Q5	
Air pollution score						
High genetic risk	Ref	1.06 (0.77, 1.47)	1.30 (0.96, 1.77)	1.07 (0.78, 1.47)	2.11 (1.59, 2.81)	0.001
Intermediate genetic risk	Ref	0.95 (0.61, 1.47)	1.41 (0.94, 2.1)	0.94 (0.60, 1.46)	2.64 (1.82, 3.81)	
Low genetic risk	Ref	1.26 (0.87, 1.82)	1.20 (0.82, 1.75)	2.05 (1.45, 2.89)	1.22 (0.83, 1.79)	
NO_2_, μg/m^3^						
High genetic risk	Ref	0.89 (0.65, 1.23)	1.19 (0.88, 1.61)	1.13 (0.83, 1.53)	1.89 (1.43, 2.50)	0.025
Intermediate genetic risk	Ref	0.75 (0.50, 1.14)	1.00 (0.68, 1.48)	0.85 (0.57, 1.28)	1.96 (1.38, 2.77)	
Low genetic risk	Ref	0.67 (0.46, 0.99)	1.14(0.82, 1.59)	1.18 (0.85, 1.66)	1.25 (0.89, 1.76)	
NO, μg/m^3^						
High genetic risk	Ref	1.29 (0.95, 1.76)	0.93 (0.66, 1.29)	1.32 (0.97, 1.8)	2.10 (1.58, 2.79)	0.014
Intermediate genetic risk	Ref	0.93 (0.60, 1.45)	1.38 (0.92, 2.06)	1.04 (0.68, 1.6)	2.46 (1.70, 3.56)	
Low genetic risk	Ref	1.43 (0.98, 2.10)	1.45 (0.99, 2.12)	1.87 (1.29, 2.7)	1.82 (1.26, 2.64)	
PM_10_, μg/m^3^						
High genetic risk	Ref	0.95 (0.70, 1.28)	0.87 (0.64, 1.18)	1.48 (1.12, 1.95)	1.53 (1.16, 2.00)	0.001
Intermediate genetic risk	Ref	2.68 (1.82, 3.95)	0.53 (0.31, 0.92)	1.92 (1.28, 2.89)	1.88 (1.25, 2.83)	
Low genetic risk	Ref	1.26 (0.87, 1.83)	1.92 (1.36, 2.69)	1.46 (1.01, 2.1)	1.05 (0.71, 1.56)	
PM_2.5_, μg/m^3^						
High genetic risk	Ref	1.29 (0.95, 1.76)	1.37 (1.01, 1.86)	1.20 (0.88, 1.64)	1.76 (1.32, 2.35)	0.019
Intermediate genetic risk	Ref	0.95 (0.61, 1.47)	1.52 (1.02, 2.26)	0.94 (0.61, 1.46)	2.45 (1.70, 3.55)	
Low genetic risk	Ref	1.42 (0.97, 2.07)	1.69 (1.17, 2.46)	1.87 (1.30, 2.70)	1.42 (0.97, 2.09)	

Model 3 was adjusted for age, sex, ethnicity (White/non-White), work (employed/unemployed), education (0–7 years, 8–10 years, 11–15 years, or 16– years), income (< 18,000/18,000–52,000/52,000–100,000/ > 100,000), physical activity (continuous), drinking status (never/past/current), smoking status (never/past/current), BMI (continuous), SBP (continuous), hypertension at baseline (yes/no), and diabetes at baseline (yes/no)

We next performed a sensitivity analysis by excluding PM_10_ from the air pollution scores; after doing so, the association between air pollution scores for NO_2_, NO, and PM_2.5_ in the highest quintile and laryngeal cancer risk remained statistically significant (Supplementary Table 3). We also performed a sensitivity analysis by excluding participants who had developed laryngeal cancer within the past 2 years; after doing so, the association between air pollution scores in the highest quintile and laryngeal cancer risk remained statistically significant (Supplementary Table 4). Furthermore, we performed a sensitivity analysis by excluding participants who had lived in their place of residence for fewer than 5 years; after doing so, the association between air pollution scores in the highest quintile and the risk of laryngeal cancer remained statistically significant (Supplementary Table 5).

## Discussion

In this large prospective cohort study, we found that an air pollution score indicating high individual or combined long-term exposure to various ambient air pollutants, namely, PM_2.5_, NO_2_, and NO, was significantly associated (after multivariable adjustment) with an increased risk of laryngeal cancer. Additionally, we identified significant interactions between air pollutants (PM_2.5_, PM_10_, NO_2_, and NO) and participants with an intermediate GRS; this interaction showed that such participants were more likely to develop laryngeal cancer than participants with a low GRS when exposed to high levels of air pollution.

To date, no studies have evaluated the association of different air pollutants with the risk of laryngeal cancer; instead, most studies have focused on the association of air pollutants with the risk of respiratory diseases, such as lung cancer. For example, a large meta-analysis by Yang et al. (Wei et al. 2014) of 21 cohort studies showed that for every 10 μg/m^3^ increase in the concentration of PM_2.5_, there was a 7.23% increase in the risk of lung cancer mortality or morbidity. Similarly, a cohort study of the effects of air pollution on nearly 313,000 people in Europe showed that long-term exposure to PM_10_ and PM_2.5_ was associated with an increased risk of lung cancer, particularly lung adenocarcinoma, with HRs of 1.22 for each 10 μg/m^3^ increase in PM_10_ and 1.18 for each 5 μg/m^3^ increase in PM_2.5_ (Shete et al. 2020).

In the current study, we found a significant joint association of air pollution and GRS with the risk of laryngeal cancer. That is, the risk of laryngeal cancer associated with air pollution scores in higher quintiles was enhanced by an intermediate GRS, suggesting that the interaction of these two factors led to a higher risk of laryngeal cancer than the sum of the risk associated with each factor alone. This shows that air pollution scores may more comprehensively reflect exposure to various air pollutants than single measure, and that people with an intermediate GRS for laryngeal cancer should be more concerned about air pollution than people with a low GRS for laryngeal cancer.

Moreover, we determined that PM_2.5_ was associated with a risk of laryngeal cancer. This is presumably due to changes in people’s employment patterns and lifestyle, and increasing atmospheric concentrations of PM_2.5_. Compared with coarse airborne PM (e.g., PM_10_), fine PM (e.g., PM_2.5_) is smaller, has a larger surface area, is more reactive, and more easily combines with toxic and harmful substances, such as heavy metal ions and microorganisms (Leikauf et al. 2020). Moreover, the low mass of fine PM particles means that they have a long residence time and long transport distance in the atmosphere, and so have a greater impact than coarse PM on human health and the quality of the air environment.

Furthermore, we found that the association between air pollution score and the risk of laryngeal cancer was significantly modified by sex, SBP, smoking status, and diabetes, consistent with the fact that lifestyle factors are recognized as the main factors affecting the occurrence of laryngeal cancer. For example, most scholars believe that smoking is the main risk factor for head and neck malignancies, such as laryngeal cancer (Eeftens et al. 2012). This may be because tobacco smoke contains many carcinogenic precursors (i.e., polycyclic aromatic hydrocarbons such as benzo[*a*]pyrene, heterocyclic amines, and nitroso compounds), which can be transformed into carcinogens in vivo. These carcinogens are electrophiles that can covalently bind to nucleophilic groups in intracellular macromolecules, such as DNA. In such cases, if DNA repair is inefficient or imperfect, dysregulation of gene expression can ensue, culminating in cellular carcinogenesis (Orsini et al. 2012).

The mechanisms by which other air pollutants increase the risk of laryngeal cancer are unclear, but several mechanisms have been proposed. PM-induced oxidative stress is considered one of the most probable mechanisms of toxicity and is thought to involve organic matter in PM being transformed into electrophilic reactive metabolites, which generate intracellular reactive oxygen species (ROS). Moreover, transition-metal ions (e.g., iron, copper, vanadium, and manganese ions) present in PM could induce production of ROS via the Fenton reaction (Desquilbet and Mariotti 2010). Increased intracellular ROS may disrupt the homeostasis of antioxidant functions and processes involving ROS in the cell, leading to increased oxidative stress and adverse effects on cellular and somatic health. This stems from ROS’ ability to directly damage cells by causing lipid peroxidation, oxidative protein modification, and DNA mutation (Yang et al. 2016), resulting in cytotoxicity and genotoxicity.

Air pollution and PM contain polyaromatic hydrocarbons (PAHs), and PAHs present on human cell-absorbed PM_2.5_ are activated by intracellular biochemical metabolism to produce ROS. A key metabolite of PAHs in the human body is benzo[*a*]pyrene diol epoxide (BPDE), which can covalently bind to the nucleophilic amino terminus of guanine in DNA to form BPDE–DNA adducts; this results in DNA damage, leading to genetic mutation and cell carcinogenesis, and is associated with tumor formation (Raaschou-Nielsen et al. 2013).

To the best of our knowledge, this was the first prospective study of various air pollutants to use an air pollution score to comprehensively examine the association between PM_2.5_, PM_10_, NO_2_, and NO and the risk of laryngeal cancer among middle- and older-aged adults using data from UKB. The strengths of our study were its large sample, prospective design, and the integration of scores for various air pollutants into an air pollution score. In addition, assessment of the contribution of genetic factors to the association between air pollution and laryngeal cancer allowed us to precisely determine the effects of air pollutants on groups of participants with different GRSs.

However, there are several limitations to this study. First, air pollution is a dynamic and complex mixture of substances containing many anthropogenic and natural pollutants with carcinogenic potential. Thus, UKB’s single measurement of air pollution at baseline did not take into account the changes in air pollution before and after registration. Second, the majority of UKB’s population is from Europe; thus, further investigation is needed to determine whether the observed associations can be applied to people from other regions and ethnic groups. Third, although we combined typical risk factors and potential confounders, residual confounding was unavoidable due to the design of the observational study. Last, the sources of air pollution data did not specify the length of outdoor exposure of the sample population; therefore, the analysis of air pollutants on the incidence of the risk of laryngeal cancer was limited.

In conclusion, we found that long-term exposure to various air pollutants, individually or jointly, was associated with an increased risk of laryngeal cancer, and that the association was more pronounced among participants who were female, were smokers, had an SBP equal to or greater than 120 mmHg, and/or had diabetes. Our findings highlight the importance of comprehensively assessing and minimizing exposures to various air pollutants and implementing good lifestyle management for preventing laryngeal cancer.

## Supplementary Information

Below is the link to the electronic supplementary material.

Supplementary file1 (DOCX 25 KB)

Supplementary file2 (DOCX 27 KB)

## Data Availability

The datasets generated during the current study are publically available.
