# Supplementary material for: Association between long-term exposure to air pollution and the risk of incident laryngeal cancer: a longitudinal UK Biobank-based study
Source: Environ Sci Pollut Res Int. 2023 Mar 28;30(20):58295–303. doi: 10.1007/s11356-023-26519-y (PMC10163128; doi:10.1007/s11356-023-26519-y)
Supplement: Supplementary file 1 — Supplementary file1 (DOCX 25 KB) [file 11356_2023_26519_MOESM1_ESM.docx]

**Supplementary Table 1** Information of genetic variants associated with laryngeal cancer in the UK Biobank study

| SNP | Chromosome | Nearest Gene | EA | OA | Beta | OR | MAF | P |
| --- | --- | --- | --- | --- | --- | --- | --- | --- |
| rs310518 | 5 | VCAN | A | G | 0.103 | 1.27 | 0.6969 | 7.00E-08 |
| rs2857595 | 6 | - | A | G | - | - | 0.3215 | 2.00E-18 |
| rs9445023 | 6 | ATF1P1, U3 | T | G | 0.120 | 1.32 | 0.2596 | 7.00E-07 |
| rs77045180 | 10 | AKR1C1 | T | G | 0.399 | 2.51 | 0.0103 | 1.00E-07 |
| rs174549 | 11 | FADS1,FADS2 | A | G | 0.385 | - | 0.2804 | 2.00E-18 |
| rs10492336 | 12 | LINC02177 | A | C | 0.149 | 1.41 | 0.1735 | 4.00E-14 |
| rs40129 | 16 | LINC02177 | G | A | 0.089 | 1.23 | 0.4089 | 4.00E-07 |
| rs142021700 | 18 | RTTN | C | T | 0.596 | 3.95 | 0.01258 | 3.00E-09 |

SNP, single nucleotide polymorphism; EA, effect allele; OR, odds ratio

**Supplementary Table 2** Pearson correlations between the individual air pollutants incorporated in the air pollution score.

| Air pollutants | NO_2_ | NO | PM_10_ | PM_25_ | Air Pollution Score |
| --- | --- | --- | --- | --- | --- |
| NO_2_ | 1.00 | 0.92^*^ | 0.50^*^ | 0.86^*^ | 0.96^*^ |
| NO | 0.92^*^ | 1.00 | 0.51^*^ | 0.84^*^ | 0.96^*^ |
| PM_10_ | 0.50^*^ | 0.51^*^ | 1.00 | 0.53^*^ | 0.59^*^ |
| PM_2.5_ | 0.86^*^ | 0.84^*^ | 0.53^*^ | 1.00 | 0.94^*^ |
| Air Pollution Score | 0.96^*^ | 0.96^*^ | 0.59^*^ | 0.94^*^ | 1.00 |

^*^Indicates p<0.001.

**Supplementary Table 3** Adjusted HRs and 95% CI for air pollution score (without PM_10_) with the risk of incident laryngeal cancer in the UK Biobank

|  | Quintiles of Air Pollution Score | | | | | HR (95% CI) per SD increase | P for trend |
| --- | --- | --- | --- | --- | --- | --- | --- |
|  | Q1 | Q2 | Q3 | Q4 | Q5 |  |  |
| Event | 31 | 37 | 48 | 49 | 75 |  |  |
| Person year | 1055837 | 1052032 | 1046720 | 1045248 | 1050096 |  |  |
| Model 1 | Ref | 1.23 (0.76, 1.98) | 1.65 (1.05, 2.59) | 1.77 (1.13, 2.78) | 2.85 (1.87, 4.35) | 1.29 (1.24, 1.35) |  |
| P |  | 0.397 | 0.029 | 0.013 | 0.001 | 0.001 | 0.001 |
| Model 2 | Ref | 1.13 (0.70, 1.83) | 1.42 (0.90, 2.23) | 1.42 (0.9, 2.24) | 2.03 (1.33, 3.12) | 1.18 (1.12, 1.24) |  |
| P |  | 0.607 | 0.132 | 0.128 | 0.001 | 0.001 | 0.001 |
| Model 3 | Ref | 1.14 (0.70, 1.83) | 1.42 (0.90, 2.24) | 1.43 (0.90, 2.25) | 2.03 (1.32, 3.12) | 1.18 (1.12, 1.24) |  |
| P |  | 0.602 | 0.130 | 0.126 | 0.001 | 0.001 | 0.001 |

Model 1 was adjusted for age, sex, ethnicity (white/non-white); model 2 further adjusted for work (employed/unemployed), education (0-7 years, 8-10 years, 11-15 years, or 16- years), income (<18000/18000-52000/52000-100000/>100000), physical activity (continuous), drinking status (never/past/current), smoking status (never/past/current); Model 3 was additionally adjusted for BMI (continuous), SBP (continuous), hypertension at baseline (yes/no), and diabetes at baseline (yes/no).

**Supplementary Table 4** Associations between air pollution score and incident laryngeal cancer by excluding laryngeal cancer cases occurred in the first 2 years of follow-up

|  | Quintiles of Air Pollution Score | | | | | HR (95% CI) per SD increase | P for trend |
| --- | --- | --- | --- | --- | --- | --- | --- |
|  | Q1 | Q2 | Q3 | Q4 | Q5 |  |  |
| Event | 27 | 32 | 44 | 43 | 70 |  |  |
| Person year | 1055469.249 | 1051337 | 1046638 | 1045108.642 | 1049539 |  |  |
| Model 1 | Ref | 1.22 (0.73, 2.03) | 1.74 (1.08, 2.81) | 1.77 (1.09, 2.87) | 3.03 (1.94, 4.74) | 1.30 (1.24, 1.37) |  |
| P |  | 0.453 | 0.023 | 0.020 | 0.001 | 0.001 | 0.001 |
| Model 2 | Ref | 1.14 (0.68, 1.90) | 1.53 (0.95, 2.48) | 1.47 (0.91, 2.39) | 2.23 (1.42, 3.51) | 1.19 (1.13, 1.26) |  |
| P |  | 0.628 | 0.083 | 0.119 | 0.001 | 0.001 | 0.001 |
| Model 3 | Ref | 1.13 (0.68, 1.89) | 1.53 (0.94, 2.47) | 1.47 (0.90, 2.39) | 2.22 (1.41, 3.49) | 1.19 (1.13, 1.26) |  |
| P |  | 0.630 | 0.084 | 0.119 | 0.001 | 0.001 | 0.001 |

Model 1 was adjusted for age, sex, ethnicity (white/non-white); model 2 further adjusted for work (employed/unemployed), education (0-7 years, 8-10 years, 11-15 years, or 16- years), income (<18000/18000-52000/52000-100000/>100000), physical activity (continuous), drinking status (never/past/current), smoking status (never/past/current); Model 3 was additionally adjusted for BMI (continuous), SBP (continuous), hypertension at baseline (yes/no), and diabetes at baseline (yes/no).

**Supplementary** **Table 5** Associations between air pollution score and incident laryngeal cancer by excluding participants who live in the current address for less than 5 years.

|  | Quintiles of Air Pollution Score | | | | | HR (95% CI) per SD increase | P for trend |
| --- | --- | --- | --- | --- | --- | --- | --- |
|  | Q1 | Q2 | Q3 | Q4 | Q5 |  |  |
| Event | 24 | 25 | 41 | 40 | 65 |  |  |
| Person year | 838858.9 | 856586.9 | 852625 | 841780.273 | 805030.9 |  |  |
| Model 1 | Ref | 1.05 (0.60, 1.84) | 1.78 (1.08, 2.95) | 1.84 (1.11, 3.05) | 3.31 (2.07, 5.3) | 1.34 (1.28, 1.41) |  |
| P |  | 0.868 | 0.024 | 0.018 | 0.001 | 0.001 | 0.001 |
| Model 2 | Ref | 0.96 (0.55, 1.68) | 1.53 (0.92, 2.54) | 1.46 (0.88, 2.44) | 2.32 (1.44, 3.75) | 1.22 (1.16, 1.29) |  |
| P |  | 0.883 | 0.099 | 0.143 | 0.001 | 0.001 | 0.001 |
| Model 3 | Ref | 0.96 (0.55, 1.68) | 1.53 (0.92, 2.54) | 1.47 (0.88, 2.44) | 2.32 (1.44, 3.74) | 1.22 (1.16, 1.29) |  |
| P |  | 0.887 | 0.098 | 0.141 | 0.001 | 0.001 | 0.001 |

Model 1 was adjusted for age, sex, ethnicity(white/non-white); model 2 further adjusted for work(employed/unemployed), education (0-7 years, 8-10 years, 11-15 years, or 16- years), income (<18000/18000-52000/52000-100000/>100000), physical activity (continuous), drinking status (never/past/current), smoking status (never/past/current); Model 3 was additionally adjusted for BMI (continuous), SBP (continuous), hypertension at baseline (yes/no), and diabetes at baseline (yes/no).

**Supplementary Table 6** Stratified analyses of the association between air pollution score and laryngeal cancer (model 3)

|  | Quintiles of Air Pollution Score | | | | | P for trend |
| --- | --- | --- | --- | --- | --- | --- |
|  | Q1 | Q2 | Q3 | Q4 | Q5 |  |
| Sex |  |  |  |  |  |  |
| Female | Ref | 0.46 (0.16, 1.35) | 3.97 (1.99, 7.89) | 1.30 (0.58, 2.91) | 3.73 (1.86, 7.49) | 0.001 |
| Male | Ref | 1.13 (0.91, 1.40) | 1.22 (0.98, 1.50) | 1.26 (1.02, 1.55) | 1.78 (1.46, 2.17) |  |
| Age |  |  |  |  |  |  |
| <60 years | Ref | 1.20 (0.80, 1.81) | 2.04 (1.41, 2.94) | 1.58 (1.09, 2.31) | 2.14 (1.50, 3.06) | 0.053 |
| ≥60 years | Ref | 1.05 (0.82, 1.35) | 1.17 (0.92, 1.49) | 1.16 (0.90, 1.48) | 1.86 (1.48, 2.34) |  |
| **BMI** |  |  |  |  |  |  |
| <25 kg/m^2^ | Ref | 1.22 (0.78, 1.90) | 1.54 (1.01, 2.34) | 1.86 (1.25, 2.79) | 3.13 (2.15, 4.55) | 1.000 |
| ≥25 kg/m^2^ | Ref | 1.04 (0.82, 1.33) | 1.35 (1.07, 1.69) | 1.08 (0.85, 1.38) | 1.49 (1.19, 1.87) |  |
| **Systolic blood pressure** |  |  |  |  |  |  |
| <120mmHg | Ref | 0.81 (0.40, 1.66) | 0.96 (0.49, 1.88) | 1.87 (1.02, 3.42) | 1.39 (0.75, 2.57) | 0.004 |
| ≥120mmHg | Ref | 1.11 (0.89, 1.38) | 1.44 (1.16, 1.77) | 1.17 (0.94, 1.46) | 1.97 (1.61, 2.41) |  |
| **Smoking status** |  |  |  |  |  |  |
| No | Ref | 0.99 (0.77, 1.27) | 1.14 (0.89, 1.45) | 0.98 (0.76, 1.26) | 1.82 (1.45, 2.29) | 0.003 |
| Yes | Ref | 1.42 (0.93, 2.15) | 2.16 (1.47, 3.17) | 2.01 (1.37, 2.94) | 2.37 (1.64, 3.42) |  |
| **Drinking status** |  |  |  |  |  |  |
| No | Ref | 0.00 (0.00, 2.159) | 4.64 (1.81, 11.9) | 1.87 (0.68, 5.17) | 3.05 (1.16, 7.99) | 0.001 |
| Yes | Ref | 1.13 (0.91, 1.39) | 1.25 (1.02, 1.54) | 1.24 (1.00, 1.52) | 1.87 (1.54, 2.27) |  |
| Diabetes |  |  |  |  |  |  |
| No | Ref | 1.10 (0.89, 1.37) | 1.33 (1.09, 1.64) | 1.21 (0.98, 1.49) | 1.78 (1.46, 2.16) | 0.036 |
| Yes | Ref | 0.78 (0.23, 2.71) | 3.02 (1.13, 8.07) | 2.68 (1.00, 7.17) | 5.36 (2.10, 13.69) |  |

Model 3 was adjusted for age, sex, ethnicity(white/non-white), work(employed/unemployed), education (0-7 years, 8-10 years, 11-15 years, or 16- years), income(<18000/18000-52000/52000-100000/>100000), physical activity (continuous), drinking status (never/past/current), smoking status (never/past/current), BMI (continuous), SBP (continuous), hypertension at baseline (yes/no), and diabetes at baseline (yes/no).
