# Supplementary material for: Association between long-term exposure to air pollution and the risk of incident laryngeal cancer: a longitudinal UK Biobank-based study
Source: Environ Sci Pollut Res Int. 2023 Mar 28;30(20):58295–303. doi: 10.1007/s11356-023-26519-y (PMC10163128; doi:10.1007/s11356-023-26519-y)
Supplement: Supplementary file 2 — Supplementary file2 (DOCX 27 KB) [file 11356_2023_26519_MOESM2_ESM.docx]

**UK Biobank**1 Study across 22 centers 502412 Participants

502237 Eligible participants with no prior laryngocarcinoma disease

456701 Eligible participants with no prior othor cancers disease

418914 Included in the analysis

Excluded (lacked NO2 NO PM10 PM25 PM25_10)

**Fig 1** Flow chart of study participants
